# Impaired Face Feature-to-Location Statistical Learning and Single-Feature Discrimination in Developmental Prosopagnosia

**DOI:** 10.3390/brainsci14080815

**Published:** 2024-08-15

**Authors:** John R. Towler, Daniel Morgan, Jodie Davies-Thompson

**Affiliations:** School of Psychology, Faculty of Medicine, Human & Health Sciences, Swansea University, Swansea SA2 8PP, UKj.davies-thompson@swansea.ac.uk (J.D.-T.)

**Keywords:** prosopagnosia, face processing, retinotopic, feature processing, face map

## Abstract

Individuals with developmental prosopagnosia (DP) experience severe face memory deficits that are often accompanied by impairments in face perception. Images of human facial features are better discriminated between when they are presented in the locations on the visual field that they typically appear in while viewing human faces in daily life, than in locations which they do not typically appear (i.e., better performance for eyes in the upper visual field, and better performance for mouths in the lower visual field). These feature-to-location tuning effects (FLEs) can be explained by a retinotopically organised visual statistical learning mechanism. We had a large group of DP participants (N = 64), a control group (N = 74) and a group of individuals with a mild form of DP (N = 58) complete a single-feature discrimination task to determine whether face perception deficits in DP can be accounted for by an impairment in face feature-to-location tuning. The results showed that individuals with DP did not have significant FLEs, suggesting a marked impairment in the underlying visual statistical learning mechanism. In contrast, the mild DP group showed normal FLE effects which did not differ from the control group. Both DP groups had impaired single-feature processing (SFP) as compared to the control group. We also examined the effects of age on FLEs and SFP.

## 1. Introduction

Developmental prosopagnosia (DP) is a neurodevelopmental disorder characterised by severe face recognition impairments and often accompanied by difficulties with face perception. Individuals with DP have normal intellectual and general cognitive functioning and an absence of low-level visual impairments [1,2,3]. Within-category object recognition difficulties are often but not always present in DP [4], and face recognition difficulties remain the largest subjective complaint. DP is typically diagnosed based on a combination of subjective self-reported complaints and impaired performance on standardised face processing tasks and diagnosed individuals are estimated to occupy the bottom 2% of the population in terms of face processing ability. Neuroscientific methods including fMRI, structural neuroimaging techniques, and scalp electrophysiology have shown that DP is associated with neuronal atypicalities primarily linked to face-selective brain regions and responses in the ventral visual stream (for details, see: [1,3]. The underlying nature of face perception difficulties in DP are under considerable investigation. The current study seeks to investigate two inter-related aspects of face processing, namely single face feature processing for isolated facial features (SFP), and feature-to-location facilitation effects (FLEs; [5]).

### 1.1. Specialised Face Processing and the Current Study

Research into what makes face processing special has received considerable research attention and most studies have focused on a holistic or configural style of expert face processing [6,7,8,9,10]. This expert style of holistic face processing has also been implicated in face recognition impairments such as DP (discussed below) and the size of holistic processing effects has been shown to be correlated with face processing ability in the typical population [11,12]. What is currently unclear is whether part-based face processing is also similarly associated with face recognition ability, and whether visual processing mechanisms other than holistic face processing might account for the differences between individuals with DP and those without face processing difficulties. To address these issues, the current investigation utilises a task from a recent study which has described a novel marker of expert face perception that we term face feature-to-location facilitation effects (or FLEs). FLEs are based on feature-to-location retinotopic tuning in the visual system [5]. Participants viewed isolated facial features without a face context (isolated eyes and mouths) which occupied different retinotopic locations in the visual field. Some images were presented at locations where these features are typically present during everyday visual experience (e.g., eyes in the upper visual field), and others were presented at atypical locations (e.g., eyes in the lower visual field). Results showed that face features were better discriminated between when they occupied typical locations as compared to atypical locations. Importantly, these findings provide evidence for a retinotopic visual learning mechanism based on visual experience. These findings are consistent with another set of research findings, that individuals have a preferred fixation location while viewing faces that varies from upper or lower fixations on the face, and that face recognition performance is best when participants fixate faces in their preferred fixation location [13,14], presumably because they can take advantage of retinotopically organised face feature maps. FLEs measure the tuning of the visual system based on an individual’s visual experience of faces, and although it has been posited that FLEs could be a hallmark of expert face perception, it is currently unknown whether the size of FLEs is systematically related to face processing ability as measured by standardised tests or implicated in face recognition disorders such as DP. Importantly, the current study seeks to replicate FLEs using the same procedure as previous work and measure face perception and memory in individuals with DP and control participants without face recognition impairments. We aim to test whether this feature-to-retinotopic location perceptual learning mechanism is implicated in developmental prosopagnosia.

### 1.2. Face Perception Impairments in Prosopagnosia

It is common for groups of individuals with DP to score below the mean of control groups in tests of face perception [15], though there is substantial variation between individuals with DP, with some having severe perceptual impairments and others showing normal performance on face perception tasks (with the average group performance around -1SD below the control mean). As previously mentioned, it is widely thought that individuals with DP have impairments in an expert style of holistic or configural face processing characterised by the entire face being encoded as a whole unit in a single glance [8], or where individual features are automatically processed in relation to the overall face context [6,7,9,10,16]. Individuals with DP perform more similarly with upright and inverted faces than neurotypical control participants who show a clear advantage for upright compared to inverted faces [15,17]. Additional evidence for impaired holistic processing in DP comes from studies that have utilised the classic part–whole procedure [7], which has shown that while control participants benefit from seeing to-be-identified facial features in the context of a whole face, individuals with DP benefit less [18]. The classic composite face illusion shows that control participants find it difficult to ignore the identity of task-irrelevant face parts when judging the identity of attended face parts (when faces are presented in their normal configuration) [6]. However, individuals with DP experience less interference from unattended parts of the face [19]. These studies suggest that holistic processing impairments are common in DP. However, it is currently unclear whether individuals with DP also have problems with single-feature-based face perception and identification. Studies on feature processing in DP do not typically test performance for perceptual discriminations between single isolated facial features, instead showing features in the context of the whole face, or with other face parts [18,19,20]. Crucially, if participants with DP have impairments with an isolated single-feature discrimination task, these difficulties would naturally be independent from any holistic face processing impairments which cannot operate on single features. If participants with DP are impaired with SFP, it would be good evidence that face recognition expertise depends not only on holistic face processing, but also part-based face processing.

### 1.3. Individual Differences, Mild Prosopagnosia, and Hypotheses

Another possibility is that FLEs are more strongly associated with face perception difficulties, and less so with face memory problems. To investigate this hypothesis, we recruited a second DP group that primarily had severe problems with familiar face recognition, but milder difficulties with other aspects of face processing (mild DP group, see Section 2 for details on diagnosis). This group is heavily under-studied (or entirely excluded from research studies) for not meeting classical prosopagnosia criteria (for more details, see [21,22]. Here, we investigate the performance of this under-studied group and ascertain whether they have SFP and FLE impairments or whether their difficulties are more restricted to familiar face memory (as suggested by [21]). If such face processing impairments are more restricted to familiar face memory, we predict that participants with mild DP will show similar performance to the control group on the feature–location task in terms of having normal FLEs and SFP. If FLEs are a key hallmark of face processing expertise, we predict that individuals with DP (and particularly those with more severe face perception difficulties) will have reduced or absent FLEs. We also predict that because holistic face processing impairments in DP are usually relatively modest, that SFP impairments are likely to be widely present in DP. Because our study recruited participants from a relatively wide range of ages, we also examined, and took steps to control for, the effect of participant age on task performance by using smaller age-matched groups, and partial correlations controlling for age.

## 2. Materials and Methods

### 2.1. Participants

Three groups (DP, mild DP and control) took part in the study (total N = 199). The DP group comprised 64 individuals with DP (47 females, *M*_age_ = 53.59, *SD* = 13.46), the mild DP group included 58 participants (49 females, *M*_age_ = 52.78, *SD* = 13.03 years) and the control group was 77 individuals (48 females, *M*_age_ = 35.44, *SD* = 16.19). Importantly, the two DP groups did not differ in their age and sex distribution. To control for potential age-related differences between our DP groups and control participants, age-matched groups were also constructed (detailed below). All participants reported normal or corrected-to-normal vision. The study was approved by the Human and Health Sciences ethical protocols for the use of human participants in research at Swansea University. All participants reported no history of neurological trauma, epilepsy, or autism diagnosis.

### 2.2. Diagnostic Criteria

We recruited a large online sample of DP participants from the Swansea Face Research group database. These individuals contacted us on the basis that they suspected that they had prosopagnosia and were seeking to find out whether their face recognition complaints qualified as prosopagnosia. We defined individuals as having DP on the basis that they reported face recognition problems in daily life, scored at least two z-scores above the mean of the PI-20, confirming the presence of a range of self-reported prosopagnosic traits, and scored less than two z-scores below the mean on at least two out of three standardised tests of face identity processing, including the Cambridge Face Perception Test (CFPT), the Cambridge Face Memory Test (CFMT), and a test of famous face recognition (FFT). We also recruited additional individuals who demonstrated both subjective and objective difficulties with face recognition but did not meet the above traditional DP diagnosis criteria. We named this group our “mild DP” group, which contained individuals who reported face recognition problems in daily life, scored at least two z-scores above the mean on the PI-20, and scored less than two z-scores below the mean on one (rather than two or more) objective test of face recognition (overwhelmingly on the FFT). These mild DP individuals have face recognition problems that are more specifically related to familiar face recognition rather than unfamiliar face perception and unfamiliar face memory. The mild DP group was compared with both the control group and the DP group.

### 2.3. Procedure

Control participants filled out consent forms and demographic information as well as two standardised face processing tasks and a questionnaire prior to completing the feature–location task. The face processing tasks included the Cambridge Face Memory Test (CFMT) which involves participants learning unfamiliar faces from three photographs and then selecting the learned faces from amongst line-ups of three faces. The task becomes progressively more difficult by introducing image and lightning changes and the additional of visual noise [23]. The Famous Face Test (FFT) involves viewing 60 images of faces of well-known celebrities one face at a time and stating the individuals name or unique identifying information about them. Participants were correct if they enter the correct name or individual identifying information and incorrect if they enter the wrong name or could not provide unique identifying information. The PI-20 is a questionnaire index of prosopagnosic traits [24]. Higher scores (/100) indicate a higher endorsement of statements related to face recognition difficulties.

DP participants had already completed the CFMT, FFT, and PI20 in a prior screening session as well as the Cambridge Face Perception Test (CFPT) which involves sorting six face images by their resemblance to a target face. Face images are morphed to varying degrees to resemble the target face to objectively quantify similarity, all images are simultaneously present during each trial of the task. Control participants did not complete the CFPT.

Criteria for being in the DP group included scoring two Z-scores above the mean on the PI-20 indicating a very high level of endorsement of prosopagnosic traits, as well as scoring two z-scores below the mean on at least two (out of three) standardised tests of face processing (CFMT, CFPT and FFT). Criteria for being included in the Mild DP Group involved scoring two Z-scores above the mean on the PI-20 indicating a very high level of endorsement of prosopagnosic traits, as well as scoring two z-scores below the mean on at least one (out of three) standardised tests of face processing (CFMT, CFPT and FFT). In all cases these individuals were impaired on the FFT. These DP and Mild DP participants were retrieved from the Face Research Swansea (FaReS) database and were invited to take part in the online study via an email containing a link to Gorilla Experiment presentation software [25]. The experiment was restricted to computers or laptops (e.g., no mobile phones or iPads, etc.).

**Figure 1:**
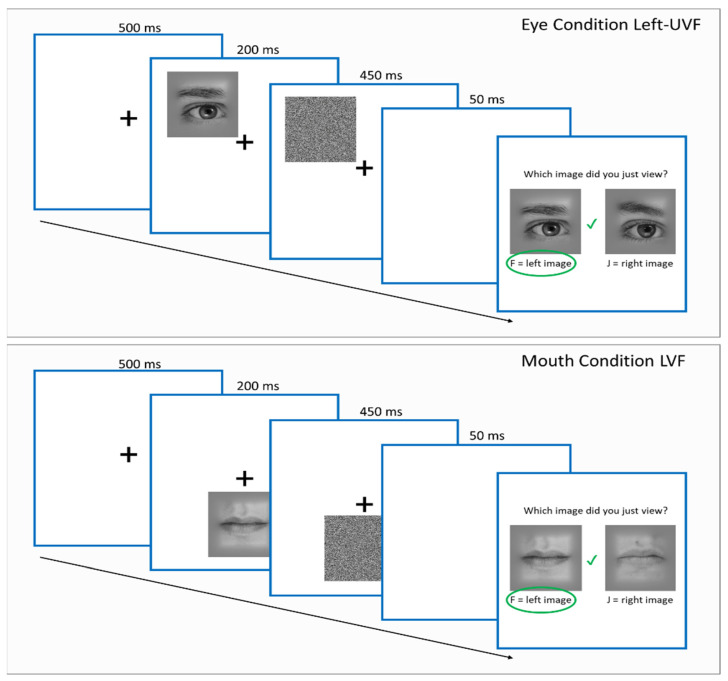
Feature discrimination task design. **Top** half of the figure represents a typical location eye condition, **bottom** half represents a typical location mouth condition. Green circle highlights the correct answer for this trial with corresponding green tick to indicate that this response was correct.

For the main feature–location task, the procedure was identical to that used in a previous study [5]. Participants were instructed to fixate on the fixation cross in the centre of the screen (corresponding to position slightly above nose to mimic natural gaze location; see, [5] for natural gaze fixation), for the duration of the experiment. Before beginning the experiment, participants also completed a short practice trial to familiarise themselves with the nature of the experiment. Following the practice trials, participants were reminded to focus on the fixation cross in the centre of the screen. The experiment began after a three second timer followed by a fixation cross in the centre of the screen on a white background. After 500 ms, a target image of a face feature appeared rapidly for 200 ms to discourage eye movements being made to the features location. This feature was then overlaid with a dynamic noise mask which lasted until 450 ms after image onset. Immediately after the noise mask, the fixation cross disappeared and two candidate test images appeared on the screen, prompting the participant to select which image had just appeared (see Figure 1). Participants responded with the F keyboard key for the image on the left, and the J keyboard key for the right image. Following their responses, participants received feedback in the form of a green tick or red cross which appeared over the fixation cross location for 200 ms, indicating whether the answer was correct or incorrect, respectively (see Figure 1). Test images were scaled versions (125% of original image size) of the target image to avoid pixel by pixel matching strategies. Importantly, there was no advantage to be acquired from fixating at any other location on the screen because face features appeared randomly and equally in the upper and lower visual fields, and upper features appeared equally to the left and right of fixation.

Participants completed 240 trials (6 blocks of 40 trials each). Each block was randomised and contained eight trials corresponding to three face features (left eye, right eye, mouth) and three stimulus positions, an upper left/right visual field (corresponding to left/right eye) and a lower visual field (corresponding to the mouth region; see Figure 1). Th eight trail types corresponded to either an eye image appearing at its ‘typical’ location (two trial types, i.e., left/right eye corresponding to an upper visual field eye condition) or the mouth location (two trial types, i.e., left/right eye corresponding to a lower visual field eye condition), and the mouth image appearing at either eye location (two trial types, i.e., left/right corresponding to an upper visual field mouth condition) and a mouth image appearing at the mouth location. To balance the design, the mouth trials were repeated in each block, yielding two trial types corresponding to a lower visual field mouth condition. The order of trials within each block was randomised. The experiment took approximately 10–12 min; participants received short breaks following each block (every 40 trials). After completing the experiment, participants read a debrief form regarding the nature of the study.

### 2.4. Stimuli

Similar to the original study [5], face feature images were based on a set of 54 frontal photographs of faces with neutral expression, none of which contained easily identifiable eye or mouth distinctive features, such as facial hair [26]. These images were used to form 27 face candidate pairs matched for gender, skin colour, and eye colour (dark/light). Each of the 27 face pairs contained a set of three face features (e.g., left eye, right eye, and mouth images). These face feature images were sampled according to a symmetric grid of squares that was overlaid on face images, such that two squares were centred on the left and right corresponding to eye regions and one centred over the mouth, corresponding to the mouth region. The respective tile regions were cut out and served as feature images in the experiment. These feature images were 466 × 466 pixels in size. The outer edge of each image was overlaid with a grey overlay which softened the edge between image and background. The participants saw 8-bit grey-scaled versions of the features that were displayed on a grey background and with a dynamic noise mask overlay (see Figure 1). The online experiment was restricted to computer monitors, participants could not complete the task on a tablet or phone. The visual angle of the images could not be reliably calculated because participants’ screen resolutions differed in size. However, importantly, we were able to ascertain that screen resolution did not predict task accuracy in the overall sample (*p* > 0.5), and that screen resolution did not reliably differ between groups (ps > 0.75). This suggests that despite differences in screen resolution, images occupied regions of the visual field (fovea/parafovea) that had adequate spatial resolution to identify the face features. We suggest that screen resolution and viewing distance are non-systematic sources of potential statistical noise in this experimental design.

## 3. Results

### 3.1. Screening Test Results

Group differences emerged for the DP and mild DP groups across all three standardised face processing tasks, but not for the PI-20 questionnaire (see Table 1). Only the DP group as a whole was classically impaired on both the CFMT and FFT (below two z-scores under the mean) and showed clear evidence of face perception difficulties on the CFPT. The DP group was significantly more impaired than the mild DP group on the CFPT, CFMT, and the FFT. The mild DP group was classically impaired on the FFT, had relatively normal performance on the CFPT, and some degree of impairment on the CFMT. All mild DP cases were diagnosed based on classically impaired performance on the FFT and PI-20, apart from three cases that were impaired on a combination of the CFMT and the PI-20. Despite differences in performance on the objective tests, both groups had comparable scores for self-reported prosopagnosic symptoms. These differences allowed us to ascertain whether SFP and FLEs were related to different degrees of face recognition impairment, and whether they are most strongly associated with face perception and unfamiliar face memory deficits, or more strongly associated with familiar face processing impairments. The control participants’ average scores were well within the normal range on the CFMT and FFT, and they did not complain of face recognition difficulties on the PI20.

### 3.2. Face Feature Discrimination Accuracy

**Figure 2:**
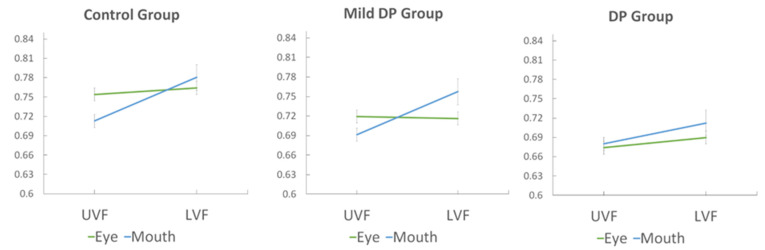
Mean proportion correct accuracy for features (eyes, mouths) and locations (upper visual field—UVF, lower visual field—LFV) in the control group, mild DP group, and DP group shown separately.

Figure 2 shows face feature discrimination accuracy (%) for the control group, mild DP group, and DP group separately for each feature type and location. In general, accuracy is higher for mouths at the typical location as compared to atypical locations; however, this feature–location effect is absent for the eyes. The mixed ANOVA with group (DP, mild DP, control), feature (eyes, mouth), and location (upper VF, lower VF) importantly revealed significant interactions between feature and location (*F*(2, 193) = 32.02, *p* < 0.001, ηp^2^ = 0.142), and feature, location, and group (*F*(2, 193) = 3.48, *p* < 0.05, ηp^2^ = 0.035). A main effect of location was also present, with discrimination accuracy being higher for lower than upper visual locations (*F*(2, 193) = 53.36, *p* < 0.001, ηp^2^ = 0.217). A main effect of group was also present, *F*(2, 193) = 16.49, *p* < 0.001, ηp^2^ = 0.146, confirming that control participants had better performance than both the DP and mild DP groups. To investigate the interaction effect with group, a series of repeated measures of ANOVAs for each of the three groups were performed, as well as a series of mixed ANOVAs comparing each group with the others. Importantly, the control group showed a significant interaction between feature and location (*F*(1, 73) = 20.43, *p* < 0.001, ηp^2^ = 0.219), confirming the presence of reliable feature–location effects; a main effect of location was also present, *F*(1, 73) = 34.35, *p* < 0.001, ηp^2^ = 0.32. In the control group, paired *t*-tests confirmed that accuracy was significantly higher for mouths at typical as compared to atypical locations, t(73) = 7.204, *p* < 0.001, and that there was no difference for eyes at typical versus atypical locations, *p* > 0.05. However, accuracy was higher for eyes than mouths in the upper visual field location, t(73) = 3.57, *p* < 0.001, providing some evidence of an eye advantage at its typical location. In contrast, the DP group showed no significant interaction between feature and location (*F*(1, 63) = 1.055, *p* > 0.05, ηp^2^ = 0.016), demonstrating an absence of reliable feature–location effects. A mixed ANOVA with DP and control groups substantiated this in a between group comparison, with a significant interaction between group, feature, and location, *F*(1, 136) = 4.399, *p* < 0.05, ηp^2^ = 0.031. The control group was more accurate overall than the DP group and the main effect of group was significant, *F*(1, 136) = 31.933, *p* < 0.001, ηp^2^ = 0.19.

The mild DP group was similar to the control group and showed a significant interaction between feature and location, *F*(1, 57) = 21.011, *p* < 0.001, ηp^2^ = 0.269. Paired *t*-tests in the mild DP group confirmed that accuracy was significantly higher for mouths at typical as compared to atypical locations, t(57) = 6.635, *p* < 0.001, and there was no difference for eyes at typical versus atypical locations, *p* > 0.05. However, accuracy was higher for eyes than mouths in the upper visual field location, t(57) = 2.156, *p* < 0.05, providing some evidence of an eye advantage at its typical location. Mixed ANOVAs comparing the mild DP to the DP and control groups separately confirmed the presence of the feature–location effects in the mild DP and control groups and the absence of these effects in the DP group. Interaction effects between feature and location (feature–location effects) in the mild DP group were significantly different from the DP group, *F*(1, 120) = 14.822, *p* < 0.001, ηp^2^ = 0.110, but not the control Group, *p* > 0.05.

### 3.3. Response Times

A mixed ANOVA with group (DP, mild DP, control), feature (eyes, mouth) and location (upper VF, lower VF) revealed a significant main effect of feature (*F*(2, 193) = 60.248, *p* < 0.001, ηp^2^ = 0.238) indicating that mouth trials were responded to more quickly than eye trials, both for when they are presented in the upper visual field (mouth upper: 1380 ms, SD = 573; eye upper: 1498 ms, SD = 648) and lower visual field (eye lower: 1485 ms, SD = 610; mouth lower: 1338 ms, SD = 566). The main effects of location and all interactions were not significant, *p* < 0.05. The main effect of group was similarly not significant, *p* > 0.05.

### 3.4. Effects of Age in Control Participants

The mean age of our DP sample is older than our control group, and our age-range within all three groups is much larger than in previous studies of the feature–location effect. Based on previous findings, we expected that age-related differences could include an impairment for processing the eyes but that other aspects of face processing may remain intact [27]. To examine and control for any potential effects of age on performance, we ran additional analyses. We split our control group into two new groups based on age (younger adults, mean age = 21.59, older adults, mean age = 49.66). Younger adults and older adults did not significantly differ on CFMT performance, FFT performance, or PI20 scores (ps > 0.05). We then ran a comparison between the younger adults and older adults (see Table 2). This analysis revealed that age decreases SFP, but only for the eyes, and at both upper and lower visual field locations. Importantly, the effect of location on mouths (FLE) remains significant in both age groups (ps < 0.05). A similar median split analysis by age for DP participants revealed no significant difference in performance for older versus younger individuals with DP (ps > 0.05).

### 3.5. Controlling for Age in Group Comparisons

To control for age as a potential confounding variable in our main DP versus control comparison, we created a new group of control participants and a new group of DP participants by matching each control participant to a DP that was closest to them in age and gender, within ±4 years of age. This created two new samples of 37 participants with DP (mean age = 49.16) and 37 controls (mean age = 49.24). Consistent with the main analysis, we found that the effect of location on mouth trials (mouth lower versus mouth upper) was significantly present for the control group, t(1, 36) = −6.634, *p* < 0.001, and the effect of location on eye trials was similarly not significant (*p* > 0.05). For the DP group, no such location effect on mouth or eye trials was present (*p* > 0.05). A mixed ANOVA on mouth trials with location (upper vs. lower) and group (DP vs. control) as factors revealed a significant interaction between mouth location and group (*F*(1, 72) = 14.57, *p* < 0.001, ηp^2^ = 0.168) confirming that there was a significant difference in the size of this feature–location effect between groups.

### 3.6. Partial Correlations

An additional step that we took to control for any potential effects of participant age was partial correlation analyses across all three groups. The rationale for these analyses was to mirror the main analyses, but to control for age as a potential confounding factor. These analyses controlled for age and were performed on accuracy scores for the face processing task battery and accuracy scores for each of the four feature–location conditions in the main feature discrimination task across all three groups (correlations involving CFPT scores did not include control participants; see Table 3).

These analyses revealed that performance on the CFMT was significantly associated with feature–location task performance at all features and locations across all groups. Within the two DP groups, the CFPT performance was associated with eye and mouth accuracy but only at their typical feature locations and not in their atypical locations confirming strong links between impaired face perception and performance with features at their typical locations. FFT accuracy was associated with feature–location accuracy at most feature and location conditions except for mouths in the upper visual field. Similarly, PI20 scores were also associated with feature–location task performance in most conditions, but not mouths in the upper visual field. Additional correlations were performed on the size of the feature–location effect for the mouth (mouth lower minus mouth upper) and these analyses showed that the size of this feature–location effect was significantly correlated with all three face processing tasks (ps < 0.05), but not with the PI20. The advantage in accuracy for eyes compared to mouths at the upper visual field location was only significantly correlated with the CFPT performance (*p* > 0.05), but not the other tasks or PI20.

These findings suggest that when controlling for participant age, both part-based feature processing and feature–location specific processing contributes to performance on these standardised face processing measures. The CFMT and FFT appear to be associated, to some extent, with both general single feature processing ability and feature-to-location effects, while CFPT accuracy appears to be related to accuracy for facial features that are present at their typical locations.

## 4. Discussion

The current study aimed to understand whether single-feature face processing (SFP) and face feature-to-location facilitation effects (FLEs) were systematically related to face recognition ability by testing control participants and two large groups of individuals with DP. We utilised a face feature discrimination task (based on [5]) in a large sample of individuals with DP and controls. Our main analysis showed that individuals with DP performed significantly worse at SFP and had significantly reduced FLEs compared to our control group, and this was confirmed by our age-matched comparisons. By comparison, our group of individuals with mild DP with familiar face processing difficulties showed no evidence for reduced FLEs but did show impairments with SFP as compared to control participants, and this was again confirmed by our age-matched control groups. With respect to our main hypotheses, we find that both single-feature processing and feature–location effects are systematically related to face recognition ability, and we discuss these findings below.

Interestingly, our experimental procedure partially replicated the results of the previous study [5]. In the original study, control participants performed better for the eyes in the upper visual field than the lower visual field, and performed better for the mouth in the lower as compared to the upper visual field. In our large control group, we replicated this effect for the mouth, but found no effect of location on the eye condition. This means that the absence of FLEs in the DP group in the current study is based on the mouth condition. The reason for this partial replication could be due to several factors. For example, it might be the case that the FLE for the mouth is simply more robust than the effect for the eyes. This is not entirely unlikely, given that stereotyped patterns of eye movements on faces tend to fixate around the bridge of the nose [5,26,28,29,30,31]. This common and stereotyped fixation behaviour means that the mouth is reliably present in the lower visual field across many kinds of fixations (eyes, nose, eyebrows, hair, etc.), in contrast, the eyes are often positioned relatively laterally to fixation rather than being clearly and reliably in the upper visual field. The original study [5] had only fourteen participants as compared to the much larger number that we report here, and it may be the case that one difference between studies is the natural tendency of participants to fixate higher up or lower down on faces (e.g., [13]), which in turn could influence the size of FLEs observed for the eyes or mouth. In any case, the presence of reliable and robust feature–location effects for the mouth demonstrates that face processing systems are tuned to the statistical regularities of natural day-to-day gaze behaviour due to a retinotopic learning mechanism which enables typical perceivers to recognise and make use of these statistical regularities (i.e., the prototypical locations of features in an upright face combined with stereotyped patterns of gaze fixation behaviour). The presence of FLEs for the mouth in the control group and the mild DP group demonstrates clear evidence for these retinotopic perceptual priors. By contrast, individuals with DP did not show evidence for this retinotopically organised face processing system. Both the age-matched groups analysis and the partial correlation analysis controlling for age confirmed and supported the results of our main analysis.

The second main finding is that single-feature processing (SFP) was impaired in our DP groups compared to control participants. To our knowledge, this is some of the first direct evidence that individuals with DP have a truly feature-based face processing deficit. These general face feature discrimination deficits were clearly observed for facial features presented in either their typical or atypical locations. This suggests that individuals with DP not only have impairments with perceptual priors involving features and locations but also more general feature-based face processing difficulties. Interestingly, the mild DP group also showed similar SFP deficits. This shows that individuals with mild DP not only have familiar face memory deficits (as assessed by the FFT), but also that perceptual deficits for single isolated facial features can be observed in this group. The mild DP group showed normal feature–location effects for the mouth, which was similar in size to the effect in the control group, suggesting that they have a normal retinotopically organised face processing system and that their perceptual impairments differed from those present in the more traditionally diagnosed individuals with DP. The results of our age-matched groups analysis and the partial correlations controlling for participant age confirmed or supported these conclusions. Overall, these findings show that single-feature face processing ability is systematically linked to face recognition ability more broadly and may be an important aspect of both normal face processing and its impairments. We also show that perceptual impairments differ between classically diagnosed individuals with DP and those with a milder form of prosopagnosia. In terms of diagnosis, the current task may be useful to help distinguish between different forms of DP that involve perceptual impairments involving individual facial features, and other forms of perceptual impairment involving feature-to-location statistical learning mechanisms.

The third finding of interest is the effects of age on face feature discrimination. We split our control group, using the median age, into older and younger control groups. We found that FLEs were unaffected by the age of control participants. However, and in contrast, we found that SFP was selectively impaired for the eyes in the older compared to younger control group adults. This deficit was observed when eyes were presented in both their typical and atypical locations. Importantly, this age-related eye-specific deficit differs from face perception difficulties experienced by individuals with DP and mild DP which were more general SFP deficits for both the eyes and mouth. Similar findings of impaired eye processing with age have been reported elsewhere [27], and our results extend these findings by showing that the ageing process impacts some aspects of face perception, but not others. It appears to be the case that eye discrimination is impaired with age, but not feature-to-location tuning or holistic face processing [27].

Here, we address potential methodological and theoretical issues. We note that, as in the original study of FLEs [5], eye movements were not directly measured during task performance. In principle, it could be the case that failure to comply with the instruction to fixate on the central cross during the task can explain the differences observed between the three groups. However, we find this unlikely for several reasons. Face features were presented at random locations (upper or lower visual field) and were presented at very rapid durations, meaning that no advantage was obtained by moving the eyes to the location of the sample feature, or by pre-emptively fixating on a potential feature location which would be unlikely to contain a face feature on a trial-by-trial basis. Additionally, there is no evidence to suggest that individuals with DP have ever had unusual difficulties following task instructions or maintaining fixation as compared to control participants. Because we have generally failed to find evidence for FLEs in the upper visual field for the eyes, it is possible that participants had a general tendency to fixate slightly above the fixation, thus reducing any potential FLEs for the eyes. However, this explanation seems unlikely, because if anything, performance is generally better for features presented in the lower visual field rather than the upper visual field. Even if this were true, it would appear to be the case across all groups tested, because no groups showed FLE for the eyes. Therefore, this slim possibility has no bearing on the significant results obtained for FLEs for the mouth, which clearly differ between groups. Furthermore, participants with mild DP showed evidence for a significant mouth FLE alongside SFP deficits, and this pattern of performance simply cannot be explained by a tendency to deviate from fixation in any way from the central cross. An additional methodological point is that due to the online nature of this experiment, screen resolution was allowed to vary freely between participants and viewing distance was not measured (participants were instructed to sit a comfortable distance from the screen). Importantly, we found that screen resolution (converted into total pixels) did not correlate with accuracy on the feature–location task, strongly suggesting that face feature stimuli fell within areas of the visual field that allow for fine-grained discriminations of visual stimuli. Additionally, screen resolution did not significantly differ between our participant groups. We suggest that these variables form potential sources of non-systematic noise in this experimental design. This methodological choice was a trade-off between experimental control and the number of participants that could attend the lab in-person. DP and mild DP participants were located across the UK and only a very small subset could feasibly come into the lab, precluding a meaningful investigation. It may be the case that the FLE for the eyes is more sensitive to variations in viewing distance and screen resolution than the FLE for the mouth, which appears to be particularly robust to these variations. Despite these potential sources of noise, we find meaningful group differences, and meaningful correlations between our feature–location task and standardised tests of face processing.

On more theoretical grounds, it is worth drawing attention to the fact that we have interpreted our findings of a reduced or absent FLE in DP as being consistent with DP participants having a deficit in a retinotopically organised face processing mechanism, implying that the learning mechanism itself is impaired in DP. However, the cause of this impairment may originate from a different source. For example, it might be the case that individuals with DP have dysfunctional patterns of gaze fixations on faces, or had these atypical gaze patterns during development, altering the spatial layout of retinoptically-organised face maps. Disproportionately fixating on features other than the bridge of the nose and other internal facial features would in turn change the spatial layout of experience-based retinotopic visual maps in these individuals. If this is the case, then it is possible that the visual learning mechanism itself may be intact in DP but tuned differently based on an atypical visual experience. For example, if an individual with DP had a strong tendency to focus attention on the hairline for recognition, then both the eyes and mouth would predominately occupy the lower visual field, or vice versa for an individual who pays a disproportionate amount of attention to (for example) the chin. Such a possibility would be consistent with previous work showing that face recognition performance is superior when individuals fixate on parts of the face that they naturally tend to look at [13,14]. Distinguishing between these two hypotheses is beyond the scope of the present report but could be investigated in future work.

## 5. Conclusions

In summary, we replicated the finding that neurotypical control participants show evidence for FLEs, suggesting the presence of a retinotopically organised face feature map in the visual cortex in control participants and those with mild DP. Our findings highlight that individuals with DP do not reliably show FLEs, suggesting underlying impairments with a retinotopically organised face map. Both the DP and mild DP groups showed evidence for general SFP impairments for both eyes and mouths, confirming that single-feature-based processing deficits are linked to wider face recognition difficulties. In contrast, the normal ageing process in control participants was shown to reduce feature discrimination accuracy specifically for the eyes. Single-feature processing may be a more important aspect of face recognition than previously acknowledged. These differing patterns of perceptual impairment confirm distinct underlying difficulties associated with prosopagnosia types and the normal ageing process in those without face processing difficulties.

## Figures and Tables

**Table 1:** Z-scores for the face processing tasks (Cambridge Face Perception Test, Cambridge Face Memory test, Famous Face Test, and PI-20) for the DP and mild DP groups, and one-way *t*-test *p*-values for paired *t*-tests comparing the two DP groups on each measure.

	CFPT	CFMT	FFT	PI-20
**DP**	−0.96	−2.78	−4.63	3.84
**Mild DP**	−0.31	−1.44	−3.75	3.71
***p*-values **	0.00003	0.00000	0.00045	0.09362

**Table 2:** Percentage correct responses for the four feature-by-location conditions in the feature discrimination task with two-way independent sample *t*-test *p*-values (rounded to two decimal places) for each group comparison for each condition in the main experimental task.

	Eye Upper	Eye Lower	Mouth Upper	Mouth Lower
**Younger Controls**	78.85	78.55	71.92	77.01
**Older Controls**	71.84	74.25	70.66	79.12
***p*-values**	0.002	0.044	0.592	0.395

**Table 3:** Correlation matrix showing partial correlations for the face processing battery (CFMT, CFPT, FFT, and PI20) and the four conditions from the feature–location task controlling for participant age. Significant correlations are in italics. Note: The control group did not complete the CFPT.

	CFPT	CFMT	FFT	PI20
**Eye Upper**	0.26	0.36	0.31	−0.28
**Eye Lower**	0.17	0.42	0.33	−0.29
**Mouth Upper**	0.06	0.20	0.11	−0.12
**Mouth Lower**	0.21	0.36	0.28	−0.19

## Data Availability

The original contributions presented in the study are included in the article; further inquiries can be directed to the corresponding author.
